# A Decision Support System for Diagnosing Diabetes Using Deep Neural Network

**DOI:** 10.3389/fpubh.2022.861062

**Published:** 2022-03-17

**Authors:** Osama Rabie, Daniyal Alghazzawi, Junaid Asghar, Furqan Khan Saddozai, Muhammad Zubair Asghar

**Affiliations:** ^1^Information Systems Department, Faculty of Computing and Information Technology, King Abdulaziz University, Jeddah, Saudi Arabia; ^2^Faculty of Pharmacy, Gomal University, Dera Ismail Khan, Pakistan; ^3^Institute of Computing and Information Technology, Gomal University, Dera Ismail Khan, Pakistan

**Keywords:** disease diagnoses, deep learning, diabetes prediction, decision support system, disease diagnosis

## Abstract

**Background and Objective:**

According to the WHO, diabetes mellitus is a long-term condition marked by high blood sugar levels. The consequences might be far-reaching. According to current increases in mortality, diabetes has risen to number 10 among the leading causes of mortality worldwide. When used to predict diabetes using unbalanced datasets from testing, machine learning (ML) classifiers and established approaches for encoding categorical data have exhibited a broad variety of surprising outcomes. Early studies also made use of an artificial neural network to extract features without obtaining a grasp of the sequence information.

**Methods:**

This study offers a deep learning-based decision support system (DSS), utilizing bidirectional long/short-term memory (BiLSTM), to accurately predict diabetic illness from patient data. In order to predict diabetes, the BiLSTM hybrid model was used after balancing the data set.

**Results:**

Unlike earlier studies, this proposed model's trial findings were promising, with an accuracy of 93.07%, 93% precision, 92% recall, and a 92% F1-score.

**Conclusions:**

Using a BILSTM model for classification outperforms current approaches in the diabetes detection domain.

## Introduction

With the emergence of AI, data mining applications have become more prevalent in several fields, such as business, education, and healthcare. Healthcare decision support systems are a hot research issue because they allow the finding of exciting patterns and useful data from enormous quantities of healthcare records. Decision support systems might help human medical specialists diagnose illnesses faster by transforming data sources into relevant insights (1). In the fast expanding discipline of data mining known as “deep learning” (DL), a complex mix of feature encoding approaches is used in order to understand from prior data to produce correct estimates (2). There are a number of uses for it, including sentiment classification (3), smart agriculture (4), and more. Recently, neural network models have shown a remarkable capability for content prediction and classification. As shown in Shickel et al. (5) and Miotto et al. (6), deep learning algorithms have figured prominently in healthcare for knowledge discovery and disease diagnosis, such as cardiac diseases, psychiatric disorders, and diabetes disorders, using health data.

### A Need for Diabetes Disease Prediction

Diabetes affects more than 34 million people in the United States, accounting for approximately 11% of the population. Diabetes is diagnosed in the United States at a rate of 17 cases per second. Each year, around 1.5 million Americans are diagnosed with diabetes (7). There are several biological indicators and risk factors that must be considered in order to get a definitive diagnosis of diabetes. Some of these indications and variables include age, gender identity, hypertension, and cholesterol levels, among others.

Following China and India, Pakistan has the third highest diabetes prevalence (8). The International Diabetes Federation (IDF) estimates that about 33 million Pakistanis have diabetes. Diabetes diagnoses are crucial since a patient's life is at stake. Diabetics must emphasize early diagnosis and treatment. Numerous complications of diabetes, including nephropathy, retinopathy, neuropathy, cardiovascular disease, stroke, and death, can be delayed or avoided with proper management of elevated blood sugar levels. More effective supervised learning algorithms for disease prediction can significantly reduce these medical errors. The healthcare sector has been supplied with a variety of supervised learning methods by researchers (9). In the health sector, data scientists are encouraged to build useful applications that may help healthcare experts diagnose and manage diabetes illness (10). To effectively predict diabetes illness, it is vital that state-of-the-art deep learning (DL) techniques be researched and applied to health-related patient data.

### The Study's Goals

In the past several years, a number of scientists have investigated the possibility of using health data to predict diabetes using computational approaches like machine learning (ML) (9, 10). This research's major goal was to discover ways to detect diabetes before symptoms appear. Additionally, they were constrained by traditional encoders that did not adequately handle the relationship among the disease dataset's predictors. Consequently, the proposed BILSTM-based framework in this research accurately diagnoses diabetes.

### Baseline Investigation

Butt et al. developed an ML-based prediction approach for diabetic disease (9). ML was used to better predict diabetes using random forest (RF), support vector machine (SVM), and other classifiers. However, ML classifiers use a traditional encoding strategy that fails to account for the predictors' underlying links. As a result, typical machine learning algorithms are often inefficient at accurately predicting diabetes risk from medical information. Because this study effort had certain limitations (9), we proposed an updated DL model called BiLSTM, which has previously been effectively employed in several fields such as DDoS attack prediction, behavior recognition, and others (2, 11). To predict diabetes, we created the BiLSTM model.

Butt et al. developed an ML-based prediction approach for diabetic disease (9). ML was used to better predict diabetes using random forest (RF), support vector machine (SVM), and other classifiers. However, ML classifiers use a traditional encoding strategy that fails to account for the predictors' underlying links. As a result, typical machine learning algorithms are often inefficient at accurately predicting diabetes risk from medical information. Because this study effort had certain limitations (9), we proposed an updated DL model called BiLSTM, which has previously been effectively employed in several fields such as DDoS attack prediction, behavior recognition, and others (2, 11). To predict diabetes, we created the BiLSTM model.

### Problem Statement

The use of traditional feature sets followed by an ML classifier makes it difficult to accurately predict diabetes from patient data (9, 10). Furthermore, the lack of relevant context makes DL models for diabetic illness prediction less effective. Predicting diabetes from patient data is treated as a binary-label prediction problem to handle the aforementioned difficulties. Diabetes is predicted from the supplied illness dataset. There are two classes of data in the dataset: D1 (yes, you have diabetes) and D2 (no, you do not have diabetes). The neural network uses these two classes to predict whether or not someone has diabetes. Using a deep neural network, we want to develop an automated system that can learn from training data and predict the presence or absence of diabetic illness using context information in the healthcare sector.

### Research Objectives

We intend to fulfill the following research goals in order to be able to perform an effective diabetic diagnosis.

RO1: To use the BiLSTM deep learning model to make predictions about diabetes based on patient illness data.
RO2: Comparison of the BiLSTM model for diabetes prediction with classical machine learning and deep learning.
RO3. Comparison of proposed method's effectiveness to baseline research for predicting diabetic patients.

### Research Contributions

The following are key contributions made by this work:

1. A deep learning (BILSTM) system is being developed to diagnose diabetic disease.
2. The proposed deep learning model for diabetes diagnosis outperforms existing classical machine learning models in terms of prediction performance.
3. To make a diabetes prognosis, two decision classes are employed.
4. Comparing the proposed strategy to existing deep learning and benchmark studies.
5. Using the proposed strategy significantly improves the model's accuracy in predicting diabetes.

The remainder of the study is organized in the following order: Section Related Work gives a review of the current literature, and Section Proposed Methodology discusses the recommended technique. Results and discussion are presented in Section Experimental Results and Discussion, and future applicability of the proposed approach is discussed in Section Conclusions and Future Work.

## Related Work

Past studies on diabetes disease prediction are summarized in this section.

Qawqzeh et al. (12) suggested a photoplethysmogram-based regression model for diabetes diagnosis. The framework was validated and evaluated using input from 450 participants and 130 pieces of information. Their suggested approach properly identified 550 non-diabetics with 92% accuracy. But the suggested approach is not compared to current methods. Automated categorization of diabetes using a machine learning technique was given by (13). They employed a SVM classifier using hyperglycemia samples from the UCI Machine Archive. It outperformed Naive Bayes, decision trees, and neural nets. While a contrast of latest systems is provided, there is no discussion of parameter estimation. An SVM-based classifier was employed by Gupta et al. (10) to identify diabetes. They made use of PIMA Indian Diabetes as a resource. Additional methods for improving predictive performance included variable selection and k-fold cross-validation. During the tests, the support vector machine did better than the naive Bayes model. In contrast, there is a lack of current comparability and consistency. Choubey et al. (14) compared numerous diabetes classification systems. The UCI Machine Learning Repository's PIMA Indian collection was integrated with an indigenous hyperglycemia collection. The researchers used SVM, KNN, and NB to identify insulin-dependent individuals from pooled datasets. PCA and LDA feature engineering approaches have been found to improve classification system performance and remove redundant features. Butt et al. (9) looked at utilizing machine learning to diagnose and forecast diabetes. It also showcases an Internet-of-things diabetic tracking device for both normal and sick people. Diabetes was classified using three classification methods: randomized forest, multilayer perceptron, and regression models (LR). They employed SVMs, MA, and linear regression to predict outcomes (LR). The study used the PIMA Indian Diabetes dataset. MLP outperforms similar learners with an accuracy of 86%, whereas LSTM outperforms others with an average of 87%. Zhou et al. (15) proposed a DTP model for glycemic control diagnosis. Each of the data sets contained over 1,000 entries. Smaller epochs in the training step ensure that the technique works rapidly on any smartphone. The findings confirm the effectiveness of the suggested model. Mujumdar and Vaidehi (16) suggested a diabetes prediction model for accurate diagnosis of diabetes that contains a few additional factors that are involved for diabetes in addition to standard indicators such as blood glucose, body mass index (BMI), age, insulin, and so on. Garca-Ordás et al. (17) introduced an algorithm based on deep learning approaches to detect diabetes patients. Variational autoencoders (VAEs) can be used to add data and features, and a CNN can be used to classify the data. Alam et al. (18) diagnosed diabetes utilizing key variables and defined their relationships. Various techniques are utilized for diabetes clustering, prognosis, and association rules. The PIMA dataset was used by Naz and Ahuja (19) to diagnose diabetes. A neural network, Multilayer Perceptron, Logistic Regression, and Deep Learning are all effective classifications that attain 90–98% efficiency. Yuvaraj and SriPreethaa (20) presented machine learning techniques in hadoop clusters for diabetes diagnosis. The results reveal that machine learning techniques can correctly predict hyperglycemia. In their study, Hasan et al. (21) built a comprehensive system for diabetes prognosis that included components such as outlier exclusion, data normalization, extraction of features, K-fold cross-validation, and several machine learning (ML) models (k-nearest neighbor, Decision Trees, Randomized Forest, Xgboost, Bayesian Network, and Gradient boosting) and Lstm. An extensive study of the uses of deep learning in diabetes was published by Zhu et al. (22). Through the exploration, a lot of original scientific papers were found. Prediction models for impaired glucose tolerance in early pregnancy were formed by Liu et al. (23). They used machine learning to make these models. The training dataset was used to build a model for predicting risk based on information gathered at registration. The deep learning classification technique makes use of the ResNet v2 CNN architecture (24), which was trained on tiny patches taken from the entire ear endoscopies before being applied to the complete ear images. A total of four deep learning models were trained for autonomous ascribable diabetic retinopathy detection, dependent on whether or not two criteria were included: DR-related lesions and diabetic retinopathy staging (25). Table 1 presents a summary of selected works.

**Table 1:** Summary of selected works.

**References**	**Technique(s)**	**Results**	**Limitations**
Butt et al. (9)	Randomized forest, multilayer perceptron, and regression models (LR)	MLP outperforms similar learners with an accuracy of 86%	Dimensionality reduction techniques not applied
Gupta et al. (10)	Support vector machine	Support vector machine did better than the naive Bayes model.	Lack of current comparability and consistency
Qawqzeh et al. (12)	Regression model	92% accuracy	Lack of comparison with the current methods
Pethunachiyar (13)	SVM classifier	Outperformed Naive Bayes, decision trees, and neural nets	There is no discussion of parameter estimation
Choubey et al. (14)	SVM, KNN, and NB	91% accuracy	Performance overhead due to incorporation of extensive feature engineering
Zhou et al. (15)	DTP model for glycemic control diagnosis	Promising results	Execution time needs to be further reduced
Garca-Ordás et al. (17)	Deep learning approach (CNN)	92.31% accuracy	Ensemble learning technique required for more better results
Alam et al. (18)	Association rules	92% accuracy	More effective preprocessing
Naz and Ahuja (19)	Multilayer Perceptron, Logistic Regression, and Deep Learning	90% accuracy	A pipeline of classifiers can produce efficient results
Yuvaraj and SriPreethaa (20)	Machine learning techniques in hadoop clusters	Outperformed baseline methods	Poor selection of predictors
Hasan et al. (21)	k-nearest neighbor, Decision Trees, Randomized Forest, Xgboost, Bayesian Network, Gradient boosting, and Lstm	LSTM exhibited better results (92% accuracy)	Parameter selection is affected by lack of efficient proper preprocessing techniques

### Existing Research Gaps

Several deep learning methods based on word embedding have previously been successful in overcoming these limitations. Using deep learning algorithms, it is important to overcome the challenge of remembering extra information in order to make highly accurate diabetes predictions.

## Proposed Methodology

**Figure 1:**
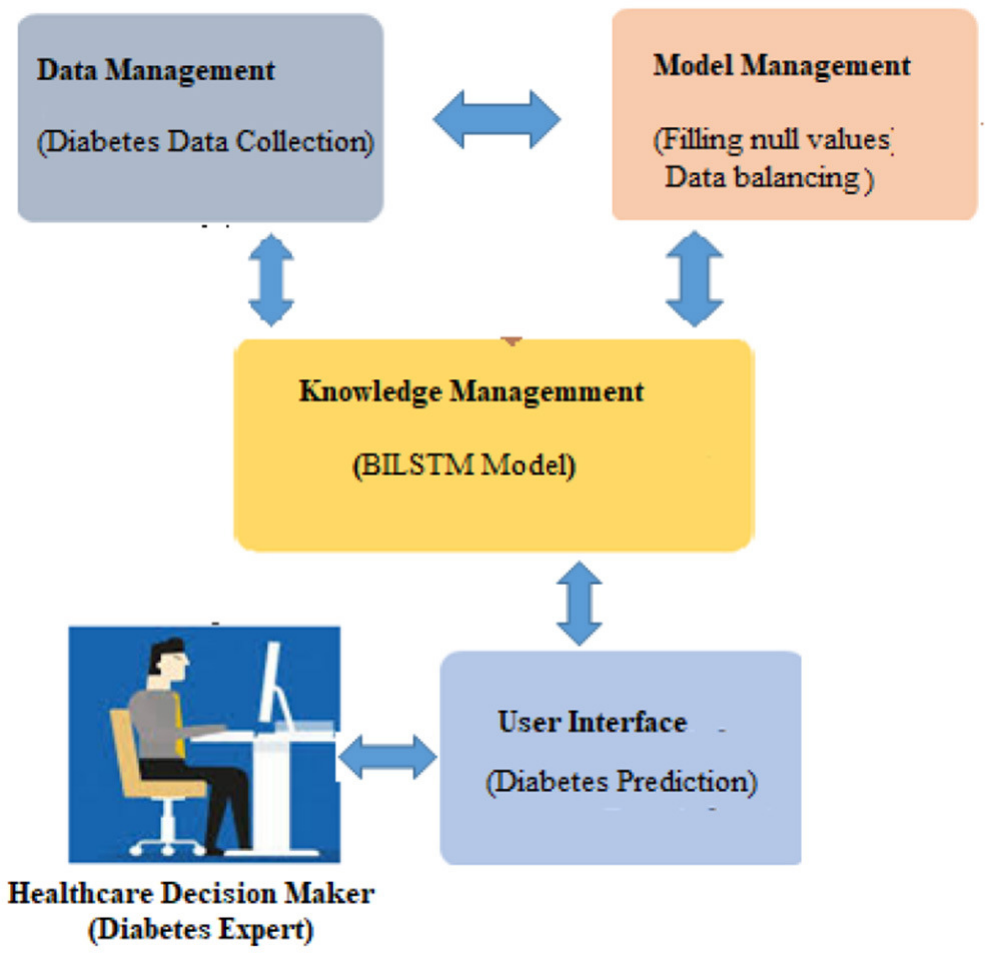
Overview of the proposed system for predicting diabetes disease.

It is vital to apply deep learning technology to incorporate current data and experience into a DSS in order to deal with this difficult decision-making challenge. Data, expertise, and models are incorporated into our DSS (see Figure 1) so that diabetic professionals may make diagnostic choices based on this information. To further involve the individuals, we sought the advice of medical specialists throughout the design process, as advised by (1). Considering the DSS's nature and the decision issue's complexity, we built the DSS according to Turban et al. (1) recommendations. The DSS has four main subsystems: data management, model management, knowledge-based management, and user interface.

### Data Management System

**Figure 2:**
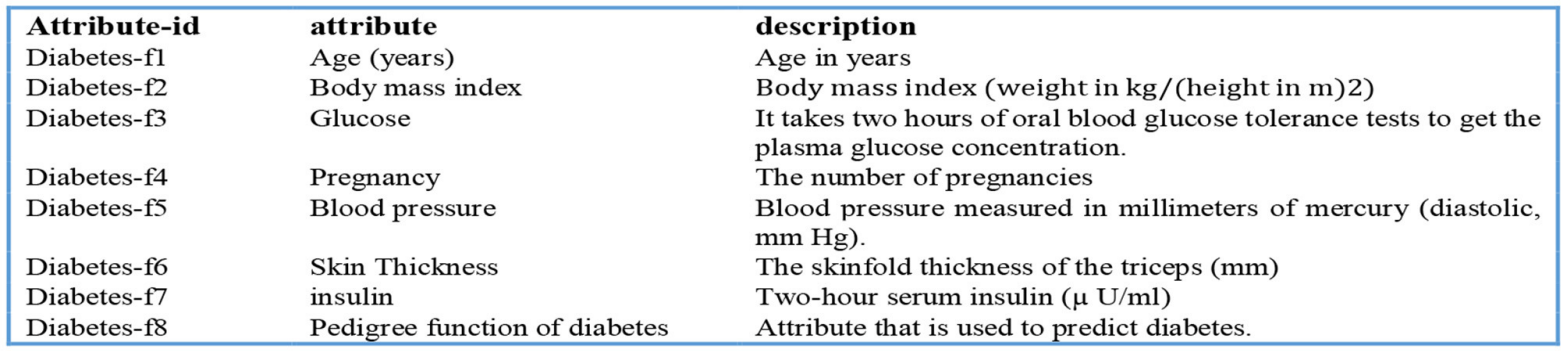
Parameters used to predict the likelihood of diabetes.

A DSS uses databases and/or datasets to provide relevant data to the decision support system. DSS data can be received from local, public, and customized sources (1), as well as institutional sources. Our decision support system's data management module collects and stores data. In this investigation, the Pima Indians' Diabetes (PID) Data Set (26) was utilized as the data source. UCI's machine learning archive does have this dataset, and it is part of a larger set of data kept by the National Institutes of Health (27). This database contains information on women of Pima Indian ancestry who were over the age of 20 at the time of data collection and who resided in the United States. The output data parameter accepts either a value of 0 or 1, with value 1 indicating a positive diabetic test and “0” indicating a negative diabetic diagnostic. An overview of the eight diagnostic attributes and their descriptions is provided in Figure 2.

#### How to Use Data

The spreadsheets are converted to CSV files. The “pd.read” command line option reads “csv”. This is a key Panda tool. We separated the training and testing sets using the sklearn training (80%) and testing (20%) partition tool (2).

#### Train Set

During training, around 80% of the training set's data was used (3). The training set includes both result identifiers (dependent variables) and input factors (predictor variables).

#### Validation Set

Using validation data in the system, efficiency concerns such as overfitting and under fitting may be addressed. Thus, a 10% validation subset is employed for model assessment (2). Both manual and automated parameter changes are possible when using Keras. With the help of automated validation in this research, a more unbiased assessment of the proposed method may be made.

#### Test Set

In order to evaluate the algorithm's efficacy, the test set includes examples that have never been seen before. This method is applied after extensive usage of the training and testing sets. The model may be evaluated using the testing dataset (11). Ten percent of the test dataset was used, which had nothing to do with the training cases. The training data set is used when the model has been fully trained. It is then checked against real data for correctness. The data is divided into 90/10 ratios by the Scikit-train-test learning division, with 10% of the data being validation data. A validation set was used to make adjustments to the model's parameters and then analyze the results.

#### Treatment of Data

The model is validated *via* 10-fold cross-validation. At each stage, we collect and keep ten replicas of the training instance. One last “holdout” model was examined in this case. We chose the version with the highest F1 score for the holdout sample.

### Model Management System

It works as a data management system. It incorporates a modelbase of statistical and other algorithms that provide sophisticated analytics to DSS. An MMS applies models to DMS data to turn it into information. In order to create a reliable prediction model, the obtained health data must be properly pretreated. The data management system handles unbalanced datasets and null value substitution.

#### Unbalanced Data Set Management

The underlying dataset is significantly imbalanced and treats both groups unevenly. Two hundred sixty-eight cases (34.9%) are present in class one for a positive test, and five hundred sixty-one cases (65.1%) are present in class zero for a negative test. Whereas, when a model learns from skewed and unequal classed data, the result usually benefits the main class whereas the minor categories are neglected in the classification stage. This is viewed as a class imbalance issue (28). To solve this issue, we use a data processing sampling method called oversampling to equalize all class instances. Oversampling is a method of expanding small classes. Random upsampling merely duplicates minor examples to increase the unbalance percentage (28). This small group replication addition considerably enhanced the classification results. In both the T1:415 and T2:414 classes, the balanced dataset is treated in the same way once random oversampling is used. Instances in total: 829.

#### Handling Missing Values

In order to increase the model's prediction performance, missing variables should be filled in with accurate data (28). In order to fill in the blanks, one can use an average, choose a random item, or go back and use the prior tier's value as a reference. After settling on the third choice, we went ahead and updated all of the missing numbers with the appropriate higher value.

### The Knowledge Management System

**Figure 3:**
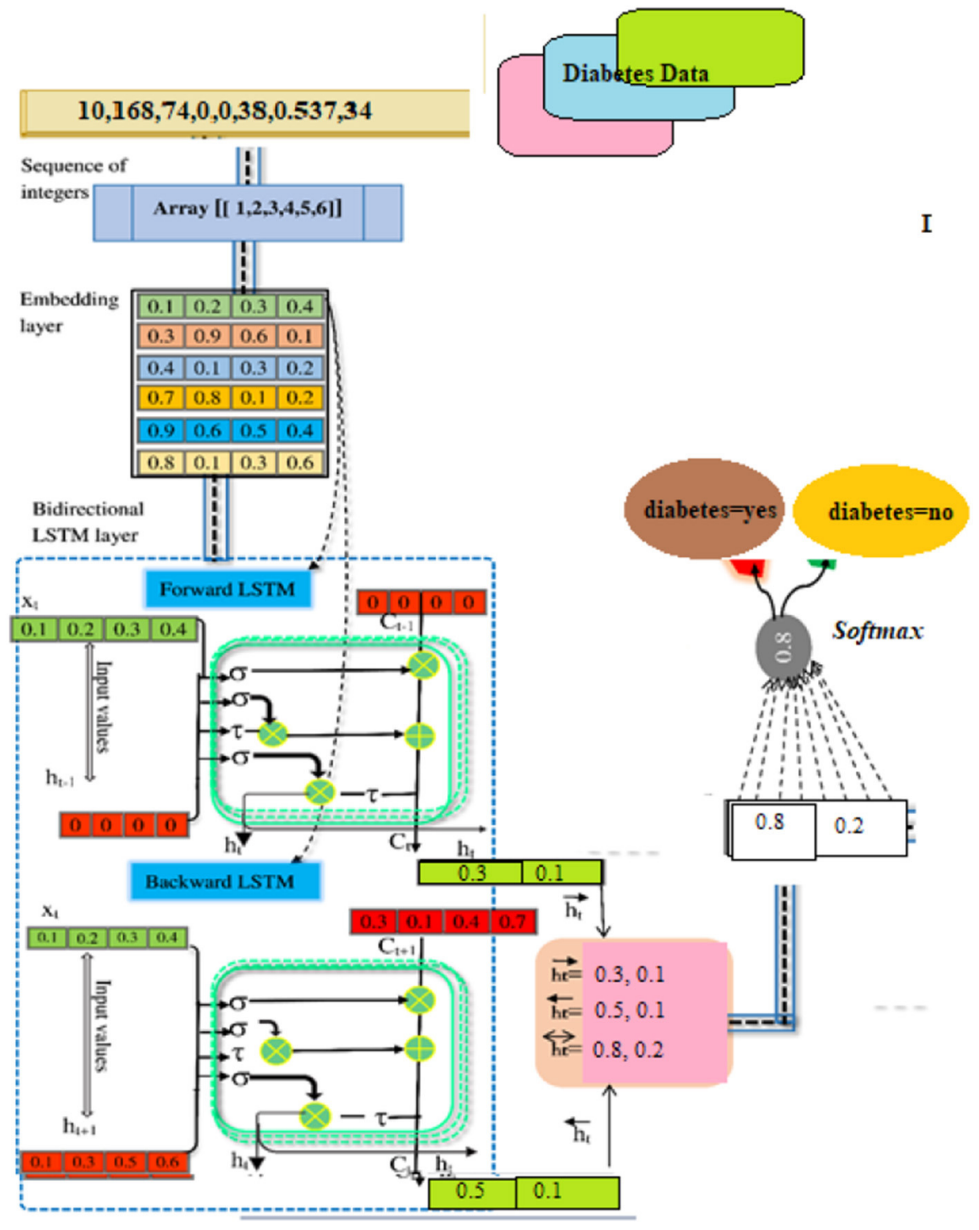
BILSTM-based for diabetes prediction system.

When used in conjunction with other DSS modules, this module may be able to provide the most up-to-date information to aid in the resolution of the issue at hand. After information has been found, acquired, and arranged, it must be transformed into knowledge. Data must be categorized, evaluated, and synthesized. The three key components of the proposed technique are: embedding layer-based data representation; bi-LSTM-based forward and backward context information saving; and softmax layer-based classification. With this numeric representation, the second module may encode features. Encoding the context of the data within a sequence using Bi-LSTM In the final module, classification is done using softmax activation (see Figure 3). Each component is described below.

#### Embedding Layer

A numeric array created by embedding items (categories) (vectors). Scalar word embeddings from discrete traits Using neural encoding to reduce attribute values and categorize has several benefits (29). Keras can embed. The diabetes dataset was encoded using attribute-level encoding matrices. Thirty-two Keras layers generate the data embedding vector. A two-dimensional embedding matrix had input data length and a word embedding dimension (feature matrix). This matrix was made by the embedding. The embedding matrices were then shifted.

#### The BILSTM Layer

The proposed method employs a deep neural network, specifically bidirectional long-short time memory (BiLSTM), to predict diabetes sickness, such as D1 (diabetes disease = yes) or D2 (diabetes disease = no). Long-term dependencies are learned using the Bi-LSTM layer. It assists in preserving the two prior and following contexts in encoded information. Instead of saving information from prior contexts, a single unidirectional LSTM only keeps data that was previously saved (30). Thus, Bi-LSTM is able to analyze encoded reviews in much more detail. Bi-LSTM uses forward and backward LSTM to learn data's past and future context (2). The following formulas are used to determine the forward and reverse LSTM:

*Formulae for forward LSTM*


(1) $$\begin{array}{l} f_{t}\;=\;\sigma \left(W_{f}x_{t}+U_{f}h_{t-1}+b_{f}\right) \end{array}$$

(2) $$\begin{array}{l} i_{t}\;=\;\sigma \left(W_{i}x_{t}+U_{i}h_{t-1}+b_{i}\right) \end{array}$$

(3) $$\begin{array}{l} o_{t}\;=\;\sigma \left(W_{o}x_{t}+U_{o}h_{t-1}+b_{o}\right) \end{array}$$

(4) $$\begin{array}{l} c\sim t\;=\;\tau \left(W_{c}x_{t}+U_{c}h_{t-1}+b_{c}\right) \end{array}$$

(5) $$\begin{array}{l} c_{t}\;=\;f_{t}⊙c_{t-1}+i_{t}⊙{c\sim }_{t} \end{array}$$

(6) $$\begin{array}{l} h_{t}\;=\;o_{t}⊙\tau \left(c_{t}\right) \end{array}$$

*Formulae for backward LSTM*


(7) $$\begin{array}{l} f_{t}\;=\;\sigma \left(W_{f}x_{t}+U_{f}h_{t+1}+b_{f}\right) \end{array}$$

(8) $$\begin{array}{l} i_{t}\;=\;\sigma \left(W_{i}x_{t}+U_{i}h_{t+1}+b_{i}\right) \end{array}$$

(9) $$\begin{array}{l} o_{t}\;=\;\sigma \left(W_{o}x_{t}+U_{o}h_{t+1}+b_{o}\right) \end{array}$$

(10) $$\begin{array}{l} c\sim t\;=\;\tau \left(W_{c}x_{t}+U_{c}h_{t+1}+b_{c}\right) \end{array}$$

(11) $$\begin{array}{l} c_{t}\;=\;f_{t}⊙c_{t+1}+i_{t}⊙{c\sim }_{t} \end{array}$$

(12) $$\begin{array}{l} h_{t}\;=\;o_{t}⊙\tau \left(c_{t}\right) \end{array}$$

#### SoftMax-Based Prediction

Afterward, SoftMax is being used to figure out how likely it is that target labels will be forecasted (i.e., the diabetes disease). The formula (Equation 18) explains how to determine the net input value


(13) $$\begin{array}{l} d_{i}\;=\;\sum w_{i}l_{i}+b \end{array}$$

“w” is the weight vector, whereas “l” stands for the input vector. “b” stands for “bias.” We can find the SoftMax by plugging it into Equation (19).


(14) $$\begin{array}{l} softmax\;\left(d_{i}\right)\;=\;\frac{expd^{d_{i}}}{\sum_{n\;=\;1}^{m}\exp^{d_{n}}} \end{array}$$

### Applied Example

We performed a number of computations to predict diabetes based on the existing disease data. The BILSTM model's every stage is discussed in detail.

#### Data Preparation

Our model predicts diabetes D1: diabetes Yes, or “D2: diabetes No” for a given patient instance in the illness dataset. Firstly, the illness data for the DL model is acquired through the instance selection module (31). The data was transformed into a matrix of indexes by a parser named Keras and transmitted to the embedding layer of the composite DL model for evaluation. The embedding layer converts each disease indicator into a vector containing streamed numbers. An example of a scalar embedding is [0.2 0.1 0.5 0.4], which encapsulates data about illness with the index [1]. In the end, the matrix packing looked somewhat like this: [0.36, 0.43, 0.85, 0.12], [0.52, 0.61, 0.11,0.25], [0.71, 0.22, 0.54, 0.47], [0.34, 0.48, 0.61, 0.39].

#### Extraction of Contextual Information

As input for this layer, a rectified feature map derived by the preceding neural network layer serves as input. In BILSTM layer calculations, the primary components are new candidate value (*c*~_*t*_), output gate (*o*_*t*_), forget gate (*f*_*t*_), and input gate (*i*_*t*_).

#### Hidden Layer No. 1

It includes the LSTM's current input (*i*_*t*_) and prior state (*h*_*t*−1_) The computations are done using Equations (1)–(6). Finally, the first hidden layer computes the “$\vec{h}$” hidden state (forward pass LSTM).


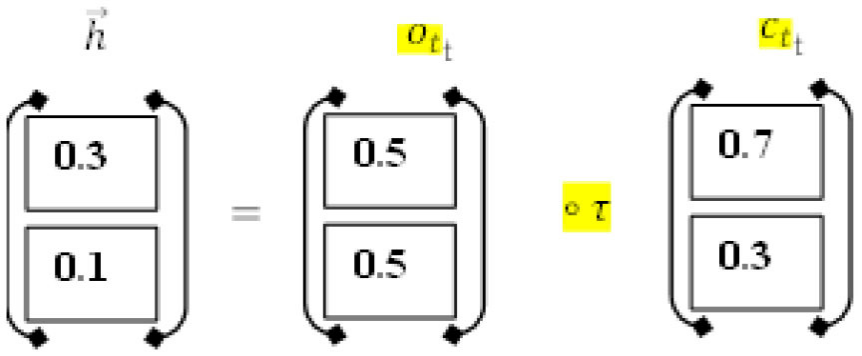


#### Hidden Layer No. 2

The present input (*i*_*t*_) and the future state (*h*_*t*+1_). make up the backward pass LSTM. The calculations are carried out by the use of the formulas (7) to (12). Finally, the succeeding layer is used to produce the hidden state $\overset{⃖}{h}$(backward pass LSTM).


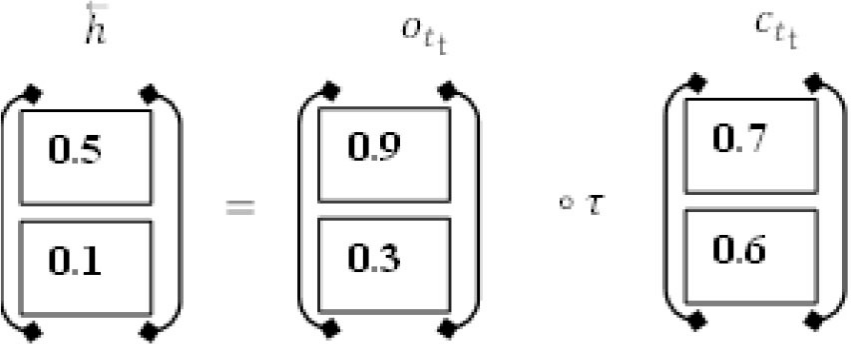


#### BISLTM Outcome

In order to get the final BISLTM “$\overset{↔}{h}$” we combine the “$\vec{h}$” from the LSTM forward pass and the “$\overset{⃖}{h}$” from the LSTM backward roll using Formula (5).


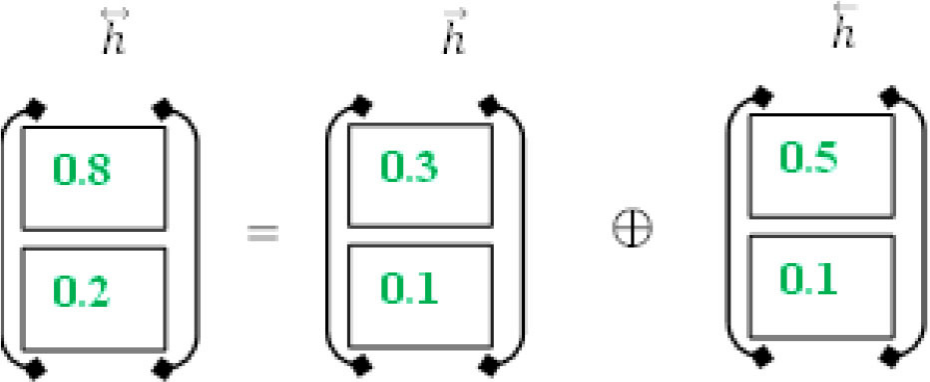


#### Diabetes Prediction

Using the SoftMax approach, it is determined how likely each of the labels “D1” and “D2” actually is. Formula (13) was used to determine the total input, as shown below:

*For diabetes-yes, the class label for decision attribute 1 is “D1.”*


$$\begin{array}{l} d_{1}\;=\;l_{1}\times w_{2}+l_{2}\times w_{2}+b\\ d1\;=\;0.7\times 0.8+0.5\times 0.2+0.5\\ d_{1}\;=\;0.56+0.4+0.5\;=\;1.46\; \end{array}$$

*For diabetes-No, the class label for decision attribute 2 is “D2.”*


$$\begin{array}{l} d_{2}\;=\;l_{1}\times w_{2}+l_{2}\times w_{2}+b\\ d2\;=\;0.4\times 0.8+0.4\times 0.2+0.5\\ d2\;=\;0.32+0.08+0.3\;=\;0.7 \end{array}$$

To figure out the likelihood of each target class (D1, D2), the SoftMax function (14) is being used.


$$\begin{array}{l} softmax\;\left(d_{1}\right)\;=\;\frac{\exp^{d_{1}}}{\sum_{i\;=\;1}^{n}\exp\left(d_{1}\right)}\\ softmax\;\left(d_{1}\right)\;=\;\frac{\exp^{1.46}}{\exp^{1.46}+\exp^{0.7}}\\ softmax\;\left(d_{1}\right)\;=\;\frac{4.305\;}{4.305+2.013}\;=\;\frac{4.305\;}{6.318}\;=\;0.681\; \end{array}$$

In the same way, the SoftMax function for the second class of diabetes predictions was made as well.


$$\begin{array}{l} softmax\;\left(d_{2}\right)\;=\;\frac{\exp^{d_{2}}}{\sum_{i\;=\;1}^{n}\exp\left(d_{2}\right)}\\ softmax\;\left(d_{2}\right)\;=\;\frac{\exp^{0.7}}{\exp^{1.46}+\exp^{0.7}}\\ softmax\;\left(d_{2}\right)\;=\;\frac{2.013\;}{4.305+2.013}\;=\;\frac{2.013\;}{6.318}\;=\;0.319\; \end{array}$$

**Figure 4:**
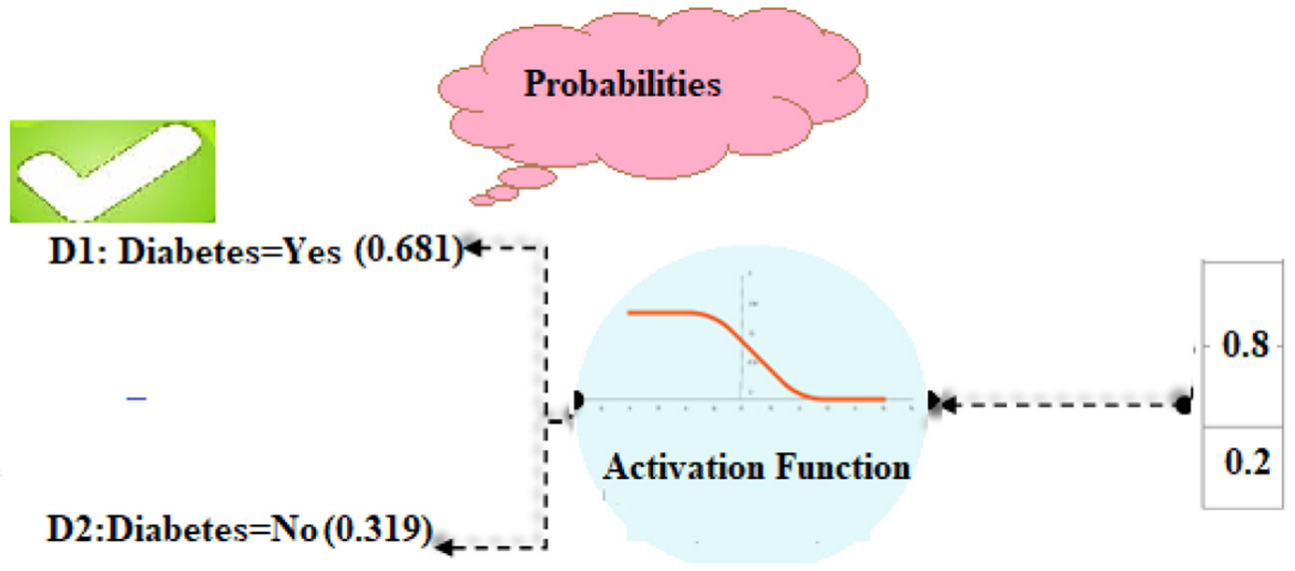
Diabetes disease classification using the softmax function.

In our computations, class D1 diabetes was shown to have the highest likelihood (0.681). Based on such patient details, we can predict that the diabetic chance is “D1” (Figure 4).

Using pseudocode, Algorithm 1 illustrates how the proposed method for forecasting diabetes illness works.

**Algorithm 1:** Methodology of the proposed diabetes disease prediction model.

A. Data Input: As a csv file, import the diabetes disease labeled dataset. B. Data Splitting: Spilt the Dataset into the Training and Testing Sets D. Vocabulary Building: Build a vocabulary for mapping disease data to integers E. Disease data stream transformation: Integerize each data stream F. Preprocessing: Carry out data preprocessing G. Data Balancing: Apply random oversampling to balance the dataset H. Model Building and Classification: Create models using classification (BILSTM) I. Performance evaluation: Prepare necessary measurements for each model to test predictive power H. Make comparisons and choose:Comparing the results and finding the best model to use.

### User Interface

The Keras package (1) provides a Python-based user interface for diabetes prediction. Diabetes forecasting software can help doctors and other healthcare providers predict diabetes. The information has been split into segments for simplicity of use and clarity of presentation. The software's body has three basic parts: data collection and preparation, classification and algorithm development, and diabetes diagnosis.

**Figure 5:**
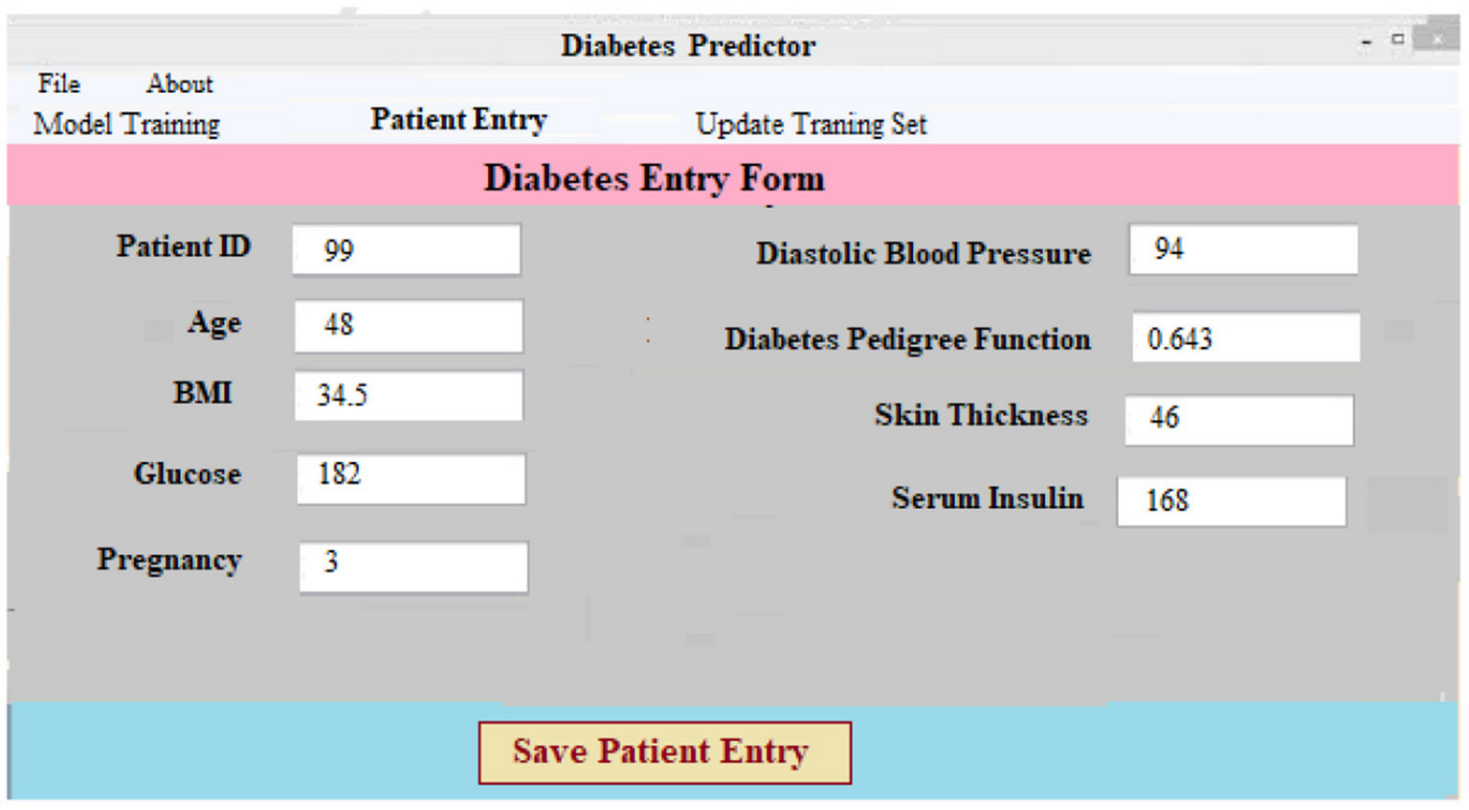
Data entry form for diabetes prediction.

*(i)* ***Data Collection and Preprocessing Component:*** This component necessitates patient information. After that, the data is preprocessed on the backend. For every patient in the database, a new case patient identity is produced with the new and better data. Using preprocessing findings, a classifier and a model to predict the result of diabetic illness are constructed. The diabetes prediction component makes predictions about a patient's ailment based on the information they've supplied. A unique patient identifier is produced as soon as the required data is entered into the system. Every patient's precise ailment is therefore identified and tracked using this identification. Figure 5 shows the screen that was used to collect and process the data for a patient registration input. At the software's server, the data is preprocessed in accordance with prior assumptions. As a result of our work, we have a layout that can be adapted to the individual requirements of each patient.

**Figure 6:**
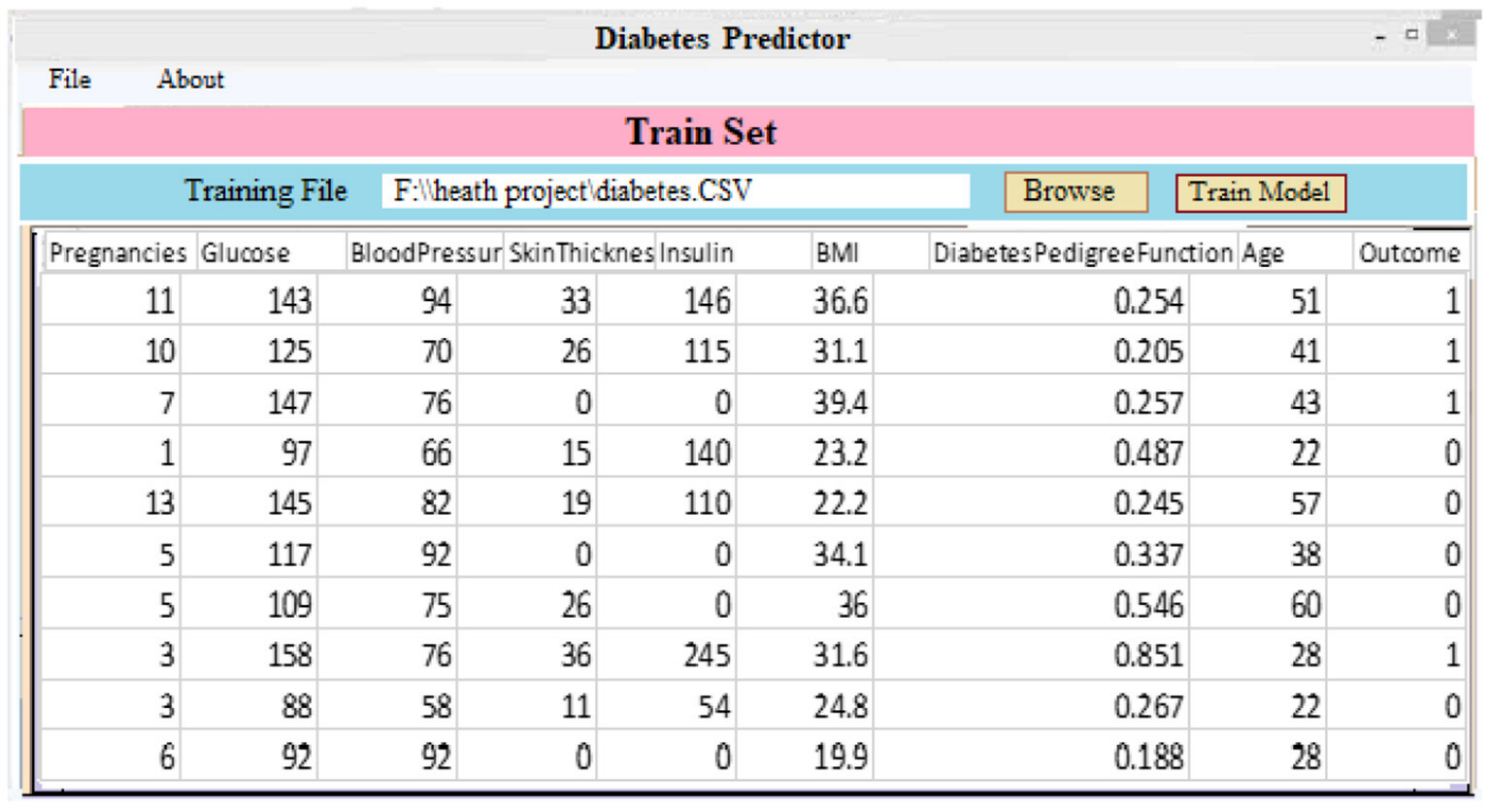
Create a model screen by loading training data.

*(ii)* ***Classifier and Model Development:*** As demonstrated in Figure 6, a diabetic dataset may be utilized to train classifiers and build models. When a patient selects the “Model Training” tab, the screen below appears. It displays the imported data for training purposes. Clicking “Model Training” creates and trains a deep learning approach.

**Figure 7:**
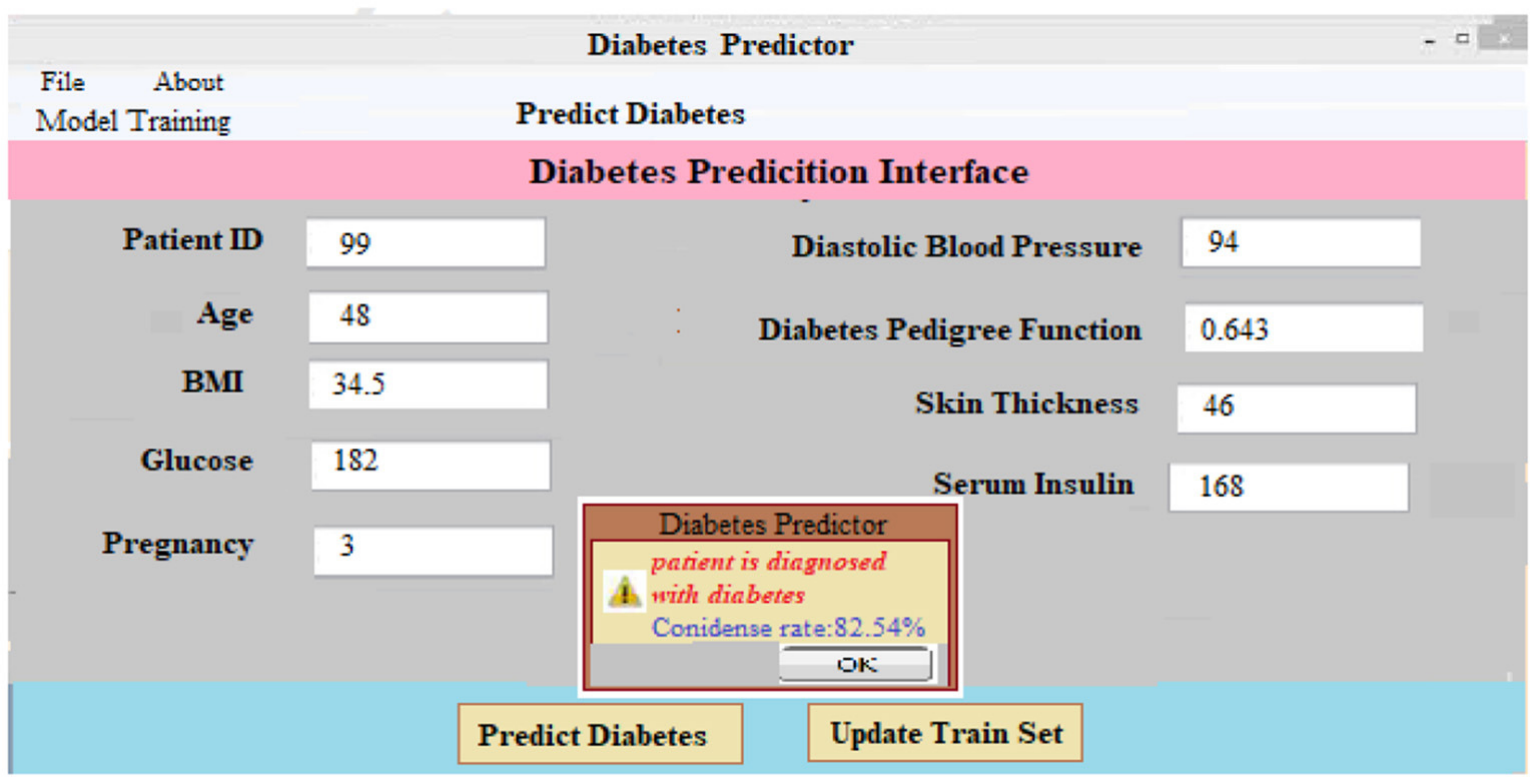
Diabetes prediction interface.

**(iii)** ***Predicting Diabetes:*** It is as simple as entering the necessary patient information and clicking the “Predict Diabetes” option on the site to predict diabetes. Whenever the “update training set” tab is pressed, a new training set is generated. After inputting the patient's disease information and pressing the “predict diabetes” button, the patient's findings are shown as “D1: diabetes Yes,” “D2: diabetes No,” and a predicted level of acceptance for each choice selected. As shown in Figure 7, the likely possibility of a diabetic illness diagnosis for a given set of criteria is “D1: diabetes Yes.”

## Experimental Results and Discussion

In this section, we will go through the findings from a series of experiments designed to address the questions posed in Section Introduction of this article.

## Addressing Research Objectives

### RO1: To Use the BiLSTM Deep Learning Model to Make Predictions About Diabetes Based on Patient Illness Data

We achieved study objective #1 by using multiple BILSTM algorithms for diabetes prediction by modifying the parameters of the recommended BILSTM model. Additionally, there were several other epochs and filtering methods in use. The algorithm has three hidden layers as well as a number of batch sizes and epochs. The number of vocabulary vectors was 62, and the embedding dimension was 128 with the SoftMax activation function being employed (8, 16, and 32). Table 2 shows the accuracy, recall, and F-score of several BILSTM models. It is 93% accurate with the following parameters: filter number 8, filer size 280, unit size 2, “f1 score” of 92%, recall of 92%, and precision of 92%.

**Table 2:** BILSTM deep learning models' accuracy, recall, and f1-score.

**Model**	**Precision (%)**	**Recall (%)**	**F-score (%)**
BILSTM-1	0.74	0.75	0.74
BILSTM-2	0.83	0.77	0.75
BILSTM-3	0.85	0.82	0.80
BILSTM-4	0.86	0.84	0.83
BILSTM-5	0.89	0.85	0.84
BILSTM-6	0.88	0.88	0.87
BILSTM-7	0.89	0.89	0.88
BILSTM-8	0.90	0.89	0.89
BILSTM-9	0.92	0.90	0.90
BILSTM-10	0.93	0.92	0.92

Data balancing greatly increases efficiency, as seen in Table 3, compared to not employing data balancing. Using the experimental data, the suggested model may be used to accurately forecast diabetes in real-world contexts.

**Table 3:** Performance of the BILSTM models with and without balancing data.

**Performance measure**	**Without data balancing**	**With data balancing**
Accuracy (%)	82	93.07
Precision (%)	82	93
Recall (%)	82	92
F1-score	88	92

Table 4 shows the results of 10 BILSTM trials with varied parameter values. We compared the accuracy of each model. The BILSTM-10 model had the greatest accuracy, with eight filter sizes, 16 filters, and 10 LSTM units.

**Table 4:** The BILSTM models' accuracy, test loss, and training time.

**Model**	**Test accuracy (%)**	**Test loss**	**Train time (s)**
BILSTM1	81.23	0.78	18
BILSTM2	82.21	0.86	5
BILSTM3	83.71	1.04	17
BILSTM4	84.67	1.13	15
BILSTM5	85.98	0.92	6
BILSTM6	87.47	0.91	13
BILSTM7	88.21	1.11	11
BILSTM8	88.36	0.80	13
BILSTM9	89.23	0.91	15
BILSTM10	92.15	0.82	10

#### Computational Complexity

Since the input data is routed *via* two LSTM layers, the computational cost of a conventional LSTM model per each stage with a gradient descent optimizer is O (W), where W is the maximum number of variables (21). However, despite the fact that BiLSTM has high computational complexity, it is successful in reducing the volume and complexity of the feature space. BiLSTM takes advantage of the data's inherent properties. Using drop out, we can cut down on the number of features and make sure the model doesn't fit even more than it should.

### RO2: Comparison of the BiLSTM Model for Diabetes Prediction With Classical Machine Learning and Deep Learning

The BILSTM results for diabetic disease prediction were contrasted with those from several traditional machine learning approaches as well as deep learning techniques in order to address the second study objective.

#### Machine Learning vs. Proposed System (BILSTM)

In order to evaluate the suggested approach (BILSTM) with other common machine learning methods, data from patients was employed. Feature representation techniques such as TF-IDF and CountVectorizer are used in machine learning. Table 5 shows the results of the performance evaluations. The results are summarized below.

- **BILSTM vs. KNN**
  To see how well the suggested BILSTM model stacks up against other machine learning techniques, we used the K-nearest neighbors approach. Table 5 shows the results of the analysis.
- **BILSTM vs. DT**
  The purpose of this experiment was to compare a BILSTM model against a traditional machine learning classifier (DT). Table 5 shows that the precision (0.81), recall (0.78), F1-score (0.80), and accuracy (0.78) of DT classifiers were all poorer (80%).
- **BILSTM vs. SVM**
  The results from the BILSTM model were found to be more effective than those from the SVM classification algorithm. Table 5 displays a lower F1-score (0.79), a lower recall (0.81), and a lower precision (0.79).
- **BILSTM vs. NB**
  BILSTM was tested against a Nave Bayes (NB) classifier in this experiment. Table 5 shows a lower F1-score (0.70%), lower recall (0.70%), and lower precision (0.70%).

**Table 5:** Machine learning classifiers vs. proposed model (BILSTM).

**ML modal**	**Accuracy (%)**	**Precision (%)**	**Recall (%)**	**F-score (%)**
KNN	78	79	80	79
DT	81	80	80	80
SVM	82	79	81	79
NB	72	70	70	70
Proposed (BILSTM)	93.07	93	92	92

#### Deep Learning vs. Proposed Technique (BILSTM)

For the purpose of accurately predicting diabetes based on patient data, the suggested method is compared to other deep learning techniques, such as long/short-term memory (LSTM), convolutional neural networks (CNN), and recurrent neural networks (RNN). Table 6 summarizes the results.

- **LSTM vs. Proposed BILSTM**
  We compared the BILSTM model's performance to that of a single LSTM model throughout this research. The LSTM model has the lowest precision, recall, F1-score, and accuracy among the models shown in Table 6.
- **CNN vs. Proposed BILSTM**
  We wanted to see if the suggested BiLSTM method outperformed the CNN model in this trial. Table 6 shows that the CNN model performed poorly in terms of accuracy, precision, recall, F1 score, and precision.
- **RNN vs. Proposed BILSTM**
  We conducted this test in order to ascertain which method was the most effective. In Table 6, the RNN model's precision, recall, F1-score, and accuracy were found to be worse than expected.

**Table 6:** BILSTM vs. other DL models.

**DL model**	**Accuracy (%)**	**Precision (%)**	**Recall (%)**	**F-score (%)**
LSTM	83.36	84	83	83
CNN	83.22	81	80	81
RNN	81.11	80	81	80
Proposed (BILSTM)	93.07	93	92	92

### RO3: Comparison of Proposed Method's Effectiveness to Baseline Research for Predicting Diabetic Patients

For the third study question, we compared the proposed BILSTM model's efficacy to similar studies. The suggested system is compared to numerous benchmarking approaches to assess its efficiency. It compares our suggested BILSTM approach to a baseline study and shows that it outperforms the latter (Table 7). An exhaustive review of published approaches is challenging for numerous reasons. With so many distinct datasets, it was difficult to compare these methods.

**Table 7:** Comparison of the BILSTM model with other studies.

**References**	**Technique**	**Results**
Butt et al. (9)	Predicting diabetic illness with machine learning	Acc: 85%
Gupta et al. (10)	Predicting diabetic illness with machine learning	Acc. 88%
Proposed (BILSTM)	Predicting diabetes with deep learning (BILSTM)	93.07

#### First Study

Butt et al. (9) proposed a supervised ML model for diabetes prediction based on patient data. Data from diabetic patients was collected using a variety of machine learning techniques. The experimental findings demonstrate that the model's performance is unsatisfactory (accuracy: 88%).

#### Second Study

A ML-based approach for diabetes prediction has been suggested. They used a range of machine learning approaches to analyze diabetes data sets. Combining a DL model with a more effective strategy for data balancing may improve the model's performance.

#### Work Proposed (Our Model)

A deep neural network is employed in the proposed DL-based diabetes prediction method. The experimental results (Table 7) show that the predictor attributes (Table 7) chosen have a significant impact on the predicted (target) variable. The main reason for our success in predicting diabetic diseases is the integration of data balancing and the BILSTM deep learning model. With the help of the BILSTM layer, context data may be preserved.

### Analyzing Results

Experts' predictions are compared to the forecast provided by the proposed technique, and the proposed method's performance is evaluated. The first 12 patients' workflows are shown in Table 8.

**Table 8:** The human expert's prognosis vs. the suggested system's.

**Suspected diabetic patient**	**Diagnosis by diabetes expert**	**Prediction by BILSTM model (proposed)**
1	Diabetes = yes	Diabetes = yes
2	Diabetes = yes	Diabetes = yes
3	Diabetes = no	Diabetes = no
4	Diabetes = yes	Diabetes = no
5	Diabetes = yes	Diabetes = yes
6	Diabetes = yes	Diabetes = yes
7	Diabetes = yes	Diabetes = yes
8	Diabetes = no	Diabetes = no
9	Diabetes = yes	Diabetes = yes
10	Diabetes = no	Diabetes = no
11	Diabetes = yes	Diabetes = yes
12	Diabetes = yes	Diabetes = yes

### Threats to External Validity

As indicated in Section Addressing Research Objectives, the suggested strategy was evaluated internally to assure model stability, and two extra datasets were acquired to externally support the proposed methodology. We collected two more datasets after conducting internal validation of the suggested technique to guarantee the validity of the strategy.

#### Dataset 2

This dataset comes from the University of Virginia School of Medicine's Department of Medicine (32). It has 1,046 occurrences in two classes. Based on comparisons with the Pima Indians Diabetes Dataset and clinical experience, we chose 12 key features from 19 initial attributes that we thought were most important to look for.

#### Dataset 3

This data set was compiled *via* responses to an internet questionnaire. There are 14 variables in the questionnaire that we came up with: age, gender, being pregnant, family history, BMI, sleeping habits, quality of sleep, snoring and other snoring-related behaviors, appetite cues, tobacco, drinks, hypertension, sugar levels, and blood sugar. Sixty-eight positive and 316 negative cases make up the dataset, which is broken down into two groups. This data set of people who live in Pakistani let us test our model in a real-world setting.

By comparing it to the multiple datasets provided in this section, we demonstrate that the proposed model for diagnosing diabetes is both effective and exact. In order to evaluate the suggested approach, classifiers are created and tested on the given data sets. On the other hand, models that have been trained on the primary dataset (dataset 1) will be tested on the two new datasets that have been set up.

Table 9 provides a summary of the findings. Both the KNN and LSTM baseline techniques were tested against the proposed model (BILSTM). An average of 82% of the compared techniques (KNN and LSTM) were outperformed by the proposed solution. This study's findings validate the proposed model and its potential to enhance classification accuracy.

**Table 9:** The external validation of the proposed method.

**Dataset**	**Precision**	**Recall**	**Accuracy**
Dataset 2	0.723 (KNN) 0.761 (LSTM) 0.855 (Proposed)	0.718 (KNN) 0.752 (LSTM) 0.819 (Proposed)	0.751(KNN) 0.766 (LSTM) 0.811 (Proposed)
Dataset 3	0.718 (KNN) 0.726 (LSTM) 0.813 (Proposed)	0.817 (KNN) 0.761 (LSTM) 0.834 (Proposed)	0.781 (KNN) 0.761 (LSTM) 0.826 (Proposed)

## Conclusions and Future Work

Because of the massive increase in healthcare content, the collection and analysis of such data to identify diabetes disease in patients has become increasingly significant. In order to do this, an effective DL-based DSS model was developed and applied. Get benchmarks for data collection, preprocess, and then use a deep neural network (BILSTM) to forecast diabetes are three components of this model. In addition, the balanced and unbalanced data sets were used in subsequent tests. A BILSTM model was also used to estimate the likelihood of diabetes developing in the future. When compared to previous attempts, the findings are positive. The proposed method does have a few apparent limitations, such as the use of embedding rather than a pre-trained model. In the future, the use of diabetes data sets from many domains (e.g., patient data from several domains) with pre-trained algorithms like word2vec or Fasttext may be investigated.

## Data Availability Statement

The raw data supporting the conclusions of this article will be made available by the authors, without undue reservation.

## Author Contributions

DA: conceptualization, supervision, project administration, and funding acquisition. DA and OR: methodology and formal analysis. FS and JA: software. DA and JA: validation. OR: investigation. MA: resources and writing—original draft preparation. FS and OR: data curation. JA: writing—review and editing. MA and JA: visualization. All authors have read and agreed to the published version of the manuscript.

## Funding

This work was funded by the Deanship of Scientific Research (DSR) at King Abdulaziz University, Jeddah, under Grant No. (611-008-D1434).

## Conflict of Interest

The authors declare that the research was conducted in the absence of any commercial or financial relationships that could be construed as a potential conflict of interest.

## Publisher's Note

All claims expressed in this article are solely those of the authors and do not necessarily represent those of their affiliated organizations, or those of the publisher, the editors and the reviewers. Any product that may be evaluated in this article, or claim that may be made by its manufacturer, is not guaranteed or endorsed by the publisher.
